# Junior doctors’ preparedness to prescribe, monitor, and treat patients with the antibiotic vancomycin in an Australian teaching hospital

**DOI:** 10.3352/jeehp.2017.14.13

**Published:** 2017-06-07

**Authors:** Cameron J Phillips, Ross A McKinnon, Richard J Woodman, David L Gordon

**Affiliations:** 1 Department of Pharmacy, Flinders Medical Centre, Bedford Park, SA, Australia; 2 School of Medicine, Flinders University, Adelaide, SA, Australia; 3 School of Pharmacy and Medical Sciences, University of South Australia, Adelaide, SA, Australia; 4 Flinders Centre for Innovation in Cancer, Flinders University, Adelaide, SA, Australia; 5 Flinders Centre for Epidemiology and Biostatistics, School of Medicine, Flinders University, Adelaide, Australia; 6 SA Pathology, Microbiology and Infectious Diseases, Flinders Medical Centre, Bedford Park, SA, Australia; 7 Division of Medicine, Flinders Medical Centre, Bedford Park, SA, Australia; Hallym University, Korea

**Keywords:** Drug monitoring, Continuing medical education, Prescriptions, Self report, Vancomycin

## Abstract

**Purpose:**

We aimed to assess the preparedness of junior doctors to use vancomycin, and to determine whether attending an educational session and being provided pocket guidelines were associated with self-reported confidence and objective knowledge.

**Methods:**

This was a 2-component cross-sectional study. A 60-minute educational session was implemented and pocket guidelines were provided. Preparedness was evaluated by a self-reported confidence survey in the early and late stages of each training year, and by continuing medical education (CME) knowledge scores.

**Results:**

Self-confidence was higher among those later in the training year (n=75) than in those earlier (n=120) in the year for all questions. In the late group, vancomycin education was associated with higher self-confidence regarding the frequency of therapeutic drug monitoring (P=0.02) and dose amendment (P=0.05); however, the confidence for initial monitoring was lower (P<0.05). Those with pocket guidelines were more confident treating patients with vancomycin (P<0.001), choosing initial (P=0.01) and maintenance doses (P<0.001), and knowing the monitoring frequency (P=0.03). The 85 respondents who completed the knowledge assessment scored a mean±standard deviation of 8.55±1.55 on 10 questions, and the interventions had no significant effect.

**Conclusion:**

Attending an educational session and possessing pocket guidelines were associated with preparedness, as measured by higher self-reported confidence using vancomycin. High knowledge scores were attained following CME; however attending an educational session or possessing pocket guidelines did not significantly increase the knowledge scores. Our findings support providing educational sessions and pocket guidelines to increase self-confidence in prescribing vancomycin, yet also highlight the importance of evaluating content, format, and delivery when seeking to improve preparedness to use vancomycin through education.

## Introduction

The ability to prescribe safely and effectively is a core requirement of doctors [1]. Concerns have been reported about how well junior doctors are prepared for prescribing [2]. A recent report by the UK General Medical Council on ‘Being Prepared’ defined preparedness for new doctors as including readiness, competence, being fit for purpose, and being fit to practice. This report stated that over 13% of junior doctors felt forced to deal with clinical problems beyond their competence or experience on a daily basis, and that antibiotics were a class of medicines that nearly 10% of doctors felt unprepared to use [3]. A number of medicines have been identified as error-prone for prescriptions by junior doctors, with antibiotics being associated with many documented errors [4]. The antibiotic vancomycin is inherently challenging to prescribe, as it requires individualisation of dosing and measurements of serum drug levels to monitor for both efficacy and toxicity [5,6]. After 50 years, however, vancomycin is still widely used and is the treatment of choice for serious infections such as methicillin-resistant *Staphylococcus aureus* [7].

In Australia, junior doctors (medical postgraduates 1–2 years and above who have not completed specialist training) undergo semi-structured teaching in public hospitals guided by the Australian Curriculum Framework for Junior Doctors. Under the domain of clinical management, this framework lists prescribing, therapeutics, and treating infections as core areas for junior doctors [8]. There is modest evidence supporting educational interventions to improve antibiotic prescribing in hospitals, which can be considered as belonging to the field of antimicrobial stewardship [9], and in general to improve prescribing by junior doctors [10]. However, no current study, to our knowledge, has evaluated the impact of educational interventions on junior doctors’ preparedness to prescribe and treat patients with vancomycin. As junior doctors perform the great majority of prescribing in teaching hospitals, the aim of this study was to assess the preparedness of this group to prescribe and monitor vancomycin, and to determine whether an educational program and the provision of pocket guidelines were associated with self-reported and objective knowledge of vancomycin prescribing.

## Methods

### Setting

The study was conducted at Flinders Medical Centre (FMC), a 580-bed government teaching hospital in Adelaide, Australia.

### Subjects

The participants of the study were junior doctors identified from the register of the Trainee Medical Officer (TMO) Unit, FMC. The potential cohorts of junior doctors available to participate comprised 72 doctors in 2012, 73 in 2013, and 74 in 2014.

### Study design

This was a cross-sectional study assessing confidence and knowledge about prescribing and monitoring vancomycin conducted between 2012 and 2014. The study comprised 2 components each year. Component 1 was a self-reported confidence survey (Supplement 1), and component 2 was an online continuing medical education (CME) module on vancomycin with knowledge assessment questions (Supplement 2). The 8 survey questions relating to self-reported confidence were analysed individually, but were first subjected to a content validity assessment to assess topic coverage via a factor analysis to determine dimensionality and an analysis using the Cronbach alpha to assess internal reliability. Content validity was assessed by 4 experts (2 pharmacists and 2 physicians). Following the adaptation of several questions, agreement was reached that the questions covered all relevant aspects of the construct. We used factor analysis, with maximum likelihood used to determine whether the questions could be considered as all relating to a single confidence domain. The 8 questions provided solutions with between 1 and 4 factors. The lowest Bayesian information criterion (BIC) was obtained for that with 2 factors (BIC= 109.7) but the solution with a single factor was very similar (BIC= 110.0). In addition, the first factor was the only one of the 4 factors with an eigenvalue greater than 1, and it alone explained 69.4% of the variability in the data, indicating that the 8 questions could be thought of as relating to a single domain. The internal reliability as assessed by the Cronbach alpha was alpha= 0.929. The knowledge component questions were also assessed for content validity by the same panel of 4 experts. The knowledge questionnaire comprised 10 multiple-choice questions, which were given equal weighting for a total score ranging from 0 to 10. Agreement on the correct answer for each question was also assessed by the experts who each took the test alone before obtaining concurrence on the correct answer.

Component 1: the self-confidence survey required respondents to use a 5-point Likert scale of (1) strongly agree, (2) agree, (3) not sure, (4) disagree, and (5) strongly disagree for a series of questions. The survey was disseminated to doctors in both the early and late part of each year. In Australia, the hospital teaching year is January to December, and the survey was disseminated and completed by participants in a 4-week period commencing in January (early in the training year) and a 4-week period running from November to December (late in the training year) in 2012, 2013, and 2014. The self-confidence survey was available to complete on paper and electronically via Survey Monkey (San Mateo, CA, USA).

Component 2: the CME and knowledge assessment were provided over 3 consecutive years during the period of June to December. The CME module was disseminated to doctors via email with a link to the online knowledge assessment, also hosted via Survey Monkey. No incentives were offered to complete the self-confidence survey or CME knowledge assessment.

The study included educational support in the form of a 60-minute, face-to-face, non-compulsory educational session and the provision of pocket guidelines (Supplement 3). The educational sessions contained core information and practical advice on prescribing and monitoring, with content selected by a multidisciplinary group of local experts. The sessions were delivered 3 times each year in an effort to capture rotating doctors. Sessions began early in participants’ training year, with repeat sessions offered mid-way through the year. The laminated pocket guidelines (6×10 cm) contained the essential features of the institutional guidelines. The pocket guidelines were disseminated at educational sessions and via the TMO Unit.

### Statistical analysis

Differences in the mean Likert scale confidence scores between the early and late training groups were assessed using the independent t-test. In addition, amongst the late in training year group alone, we also compared the difference in mean confidence scores for those who attended an educational session and those who did not, and also the difference in mean confidence scores for those who possessed pocket guidelines and those who did not. The differences in the proportion of respondents correctly answering CME knowledge questions according to vancomycin prescribing experience, attendance at an educational session, and possession of pocket guidelines were assessed in a univariate analysis using the Fisher exact test. In addition, we also assessed whether these 3 factors were independent predictors of a correct response using multivariate binary logistic regression. Finally, multivariate linear regression was used to assess whether any of these 3 factors predicted total knowledge scores, as defined by the number of correct responses across the 10 questions.

### Sample power

We had 80% power to detect a difference in Likert scale confidence scores of 0.4, assuming a standard deviation in confidence for each question of 1.0 for each group (n= 120 and n= 75). In regard to assessing differences in the proportion of correctly answered CME knowledge questions, we had 80% power to detect a difference of 18%, assuming that approximately 80% of subjects correctly answered each question between 2 groups of size n= 27 and n= 58 (pocket guidelines groups) and 82% power for 2 groups of size n= 40 and n= 45 (educational session groups) for those who completed the CME knowledge questions. All analyses were performed using IBM SPSS ver. 22.0 (IBM Corp., Armonk, NY, USA).

### Ethical approval

This study received full ethics approval from the Southern Adelaide Clinical Human Research Ethics Committee, Australia (approval 123.12).

## Results

### Component 1: self-reported confidence survey

A total of 195 completed surveys were received over 2012–2014 (120 from the early group and 75 from the late group). Raw data are available in Supplement 4. The work position and experience of junior doctors is presented in Table 1. Self-reported confidence in prescribing vancomycin improved across the 8 domains between the early and late groups (P< 0.001) (Table 2).

The association of attending an educational session with self-reported confidence in prescribing vancomycin was evaluated in respondents who responded late in their year of training (n=75). Those who had attended an educational session had a higher degree of confidence in terms of knowing how often blood levels of vancomycin should be taken once the therapeutic target range is attained; Likert score, mean (95% confidence interval [CI]) 2.2 (2.0–2.4) versus 2.5 (2.1–2.9); (P= 0.02). There was a trend for significance in interpreting high or low vancomycin levels to amend the dosing among those who attended the educational session, with a mean score (95% CI) of 2.1 (1.8–2.3) versus 2.5 (2.1–2.9); (P=0.05). Surprisingly, respondents were more confident knowing when to take the first blood level of vancomycin if they had not attended an educational session, with a mean score (95% CI) of 1.9 (1.8–2.1) versus 2.4 (2.1–2.8); (P≤ 0.005) while there were no significant differences for the other remaining questions (Table 3). The association of possessing pocket guidelines with self-reported confidence in prescribing vancomycin was also evaluated in respondents from the late group (n=75). Those who possessed pocket guidelines had a higher degree of confidence in terms of treating patients with vancomycin, choosing an initial dose, choosing a maintenance dose, and knowing how often to take vancomycin blood levels (Table 4).

### Component 2: continuing medical education knowledge assessment

Preparedness to prescribe, monitor, and treat patients was determined by knowledge scores obtained after completion of an online CME module on vancomycin. Eighty-five respondents completed the CME questions. Demographic factors and experience prescribing vancomycin are presented in Table 5. The mean and standard deviation for the total knowledge score was 8.55± 1.55 from a maximum achievable score of 10. Scores were not influenced by prescribing experience, attending an educational session, or possession of the pocket vancomycin guidelines (Table 6). In multivariate linear regression, there were no significant effects for experience prescribing vancomycin (β= 0.09± 0.36, P= 0.82), attending an educational session (β= −0.56± 0.35, P= 0.12) or possessing pocket guidelines (β= 0.62± 0.38, P= 0.11). The range of correctly answered individual knowledge questions is presented in Table 6, with no differences observed for those who attended a prior educational session, were in possession of pocket guidelines, or had more experience prescribing vancomycin. However, in multivariate logistic regression, the odds of correctly answering the question about the loading dose were unexpectedly lower than in those with more experience prescribing vancomycin than in those with less experience (odds ratio [OR], 0.17; 95% CI, 0.04 to 0.81; P= 0.02), and was higher for those with a pocket guide than for those without (OR, 6.3; 95% CI, 1.11 to 36.2; P= 0.04). The odds of correctly answering the question related to managing initially elevated vancomycin levels were lower for those who attended the educational session than for those who did not (OR, 0.20; 95% CI, 0.05 to 0.83; P= 0.03). Experience prescribing vancomycin, prior attendance of an educational session, and possessing pocket guidelines were not predictors of a correct response for any of the other questions.

## Discussion

This study examined a pertinent topic with a pragmatic design employing educational interventions in the challenging environment of an authentic clinical context. During this study, we observed that junior doctors’ self-reported confidence was higher for all questions when asked later in the hospital teaching year. While it could be argued that increased confidence occurs simply with increasing experience over the year spent working as a doctor, some two-thirds of doctors reported very limited experience, having prescribed vancomycin as little as 10 or fewer times. In the current study, those doctors who had attended an educational session were more confident in the domain of therapeutic drug monitoring of vancomycin; specifically, knowing when to measure blood levels and how frequently to monitor them once the patient is in the target range, and borderline significance was found for confidence in the more complex task of interpreting vancomycin blood results to amend dosing. These are important findings, as measuring blood levels at the wrong time and frequency can result in misinterpretation of the results and lead to incorrect dosage adjustment [11]. As our educational intervention was multifaceted, we are unable to determine the effect of individual components; thus, we cannot say if future resources should be directed to face-to-face sessions, online CME, or provision of pocket guidelines. The CME with knowledge questions was developed with considerable input from pharmacy, infectious diseases, and clinical pharmacology to ensure that the CME content was contemporary. The time required to prepare the CME with questions was significantly in excess of the time required for preparation and delivery of the face-to-face sessions, yet the CME, once prepared, can be disseminated to a large audience if required. Getting junior doctors to take time out of their busy schedule for an educational session is challenging, but during an internal evaluation of these sessions, junior doctors overwhelmingly agreed or strongly agreed that attending the sessions was useful to their clinical practice.

The junior doctors who received pocket guidelines were significantly more confident on 6 of the 8 questions about dosing and monitoring vancomycin, suggesting strongly that provision of the pocket guidelines improved their confidence. These finding are meaningful, as a systematic review found that low levels of confidence had a negative impact on the preparedness of junior doctors, as did deficiencies in areas such as prescribing [12]. Furthermore, perceived confidence has been reported to have a significant effect on the clinical behaviour of medical graduates [13].

The proportions of correct responses to the online CME knowledge questions on vancomycin were generally very high, for all but 1 question regarding the management of red man syndrome. Interestingly, pharmacists have also scored low on formal CME questions about the management of red man syndrome [14]. Experience in prescribing vancomycin, attendance at a prior educational session, or possessing pocket guidelines did not increase the total knowledge scores. The multivariate analysis of answers to individual CME questions provided some unexpected findings. Those with more experience prescribing vancomycin had a lower likelihood of answering the question about the loading dose correctly than those with less experience. Similarly, the question about managing elevated vancomycin levels produced a surprising result. Those who attended an educational session were paradoxically less likely to answer this question correctly than those who did not. This counter-intuitive performance may potentially be explained by the possibility that those with more experience were less inclined to consult the guidelines for advice before prescribing. Alternatively, the educational content for these areas may have been unclear or potentially confusing, or it may be that doctors who chose to attend the educational sessions did so because they felt less informed than those who did not attend. Nevertheless, these findings emphasise the importance of careful review of the content, format, and delivery of educational interventions, as well as the need for those with educational expertise and content knowledge to evaluate them rigorously.

There are some limitations to this study. As it was cross-sectional in design, we cannot infer causality. In particular, the differences observed for those attending or not attending the educational session and those with and without pocket guidelines may be due to reverse causality. This study did not assess actual vancomycin prescriptions written by these doctors, so we do not know if their clinical behaviour changed after the educational intervention. Further studies assessing educational support to improve junior doctors’ preparedness to clinically use vancomycin should involve the rigorous evaluation of such interventions, using more programmatic approaches implemented across multiple sites with numerous sources of stakeholder input to avoid the constraints of self-reported data, as has recently been proposed by others [15]. Larger sample sizes are required to detect some of the smaller but non-significant improvements in knowledge that were observed in the study. Noteworthy, however, is that our study contained more subjects than many of the randomised and non-randomised studies included in a systematic review on educational interventions to improve prescribing for junior doctors [10].

In conclusion, to our knowledge, this is the first evaluation of junior doctors’ preparedness to prescribe the antibiotic vancomycin. Possession of pocket guidelines was associated with significantly higher self-reported confidence to use vancomycin, while attending an educational session was associated with higher self-reported confidence to perform therapeutic drug monitoring. Generally high knowledge scores were obtained by those completing an online CME assessment on vancomycin. However, no apparent effect on knowledge scores was associated with attending an educational session, possessing pocket guidelines, or having increased experience prescribing vancomycin. Based on our findings, future initiatives to improve the preparedness of junior doctors to prescribe vancomycin could include education and the provision of pocket guidelines; however, careful design and close evaluation of educational content, usability, and format require the utmost consideration.

## Figures and Tables

**Table 1.** Position of junior doctors and their experience prescribing vancomycin by training period (early versus late)

Junior doctors' characteristics	Early in training year (n = 120)	Late in training year (n = 75)	P-value^a)^
Hospital position			0.83
PGY1	115 (95.8)	73 (97.3)	
PGY2	3 (2.5)	2 (2.7)	
Other	2 (1.7)	0	
How many times prescribed vancor ycin			<0.001
≤10 times	108 (90.0)	49 (65.3)	
11-20 times	9 (7.5)	16 (21.3)	
21-30 times	1 (0.8)	7 (9.3)	
>30 times	0	3 (4.0)	
Missing	2 (1.7)	-	

Values are presented as number (%).

PGY, postgraduate year.

a) Early in the training year versus late in the training year, using the Fisher exact test.

**Table 2.** Junior doctors’ mean self-reported confidence scores by training period (early versus late)

Confidence domains “do you feel confident to”	Early in training year (n = 120)	Late in training year (n = 75)	P-value^a)^
Treat patients with VAN?	3.2±0.98 (3.1-3.4)	2.3±0.76 (2.1-2.5)	< 0.001
Choose an initial VAN dose?	3.1 ±0.99 (2.9-3.3)	1.9±0.67 (1.8-2.1)	< 0.001
Choose a maintenance VAN dose	3.1 ±0.88 (3.0-3.3)	2.0±0.74 (1.9-2.2)	< 0.001
Know when the first blood level of VAN should be measured?	3.2 ±0.90 (3.0-3.3)	2.1 ±0.70 (2.0-2.3)	< 0.001
Know how often blood levels of VAN should be taken once the patient has reached therapeutic range?	3.3±0.87 (3.1-3.4)	2.26±0.73 (2.1-2.4)	< 0.001
Know the target therapeutic range for VAN?	3.1 ±1.1 (2.9-3.3)	2.1 ±0.81 (1.9-2.3)	< 0.001
Interpret high or low VAN levels to use that information to amend the dose or interval?	3.2±0.94 (3.0-3.3)	2.2±0.91 (2.0-2.4)	< 0.001
Manage an infusion-related reaction to VAN (red man syndrome)?	3.8±0.98 (3.6-4.0)	3.2±1.1 (2.9-3.4)	< 0.001

Values are presented as mean ± standard deviation (95% confidence interval). Likert score 1 = strongly agree, 2 = agree, 3 = not sure, 4 = disagree, 5 = strongly disagree.

VAN, vancomycin.

a) Early in the training year versus late in in the training year, using the unpaired t-test.

**Table 3.** Comparison of self-reported confidence scores between those who did and do not attend a face-to-face vancomycin educational session (late in the training year only)

Confidence domains “do you feel confident to”	Did not attend prior educational session (n = 25)	Attended prior educational session (n = 49)	P-value^a)^
Treat patients with VAN?	2.5±0.9 (2.1-2.9)	2.2±0.7 (2.0-2.4)	0.09
Choose an initial VAN dose?	2.0±0.6 (1.7-2.3)	1.9±0.7 (1.7-2.1)	0.47
Choose a maintenance VAN dose	2.2±0.9 (1.8-2.6)	1.9±0.7 (1.7-2.1)	0.15
Know when the first blood level of VAN should be measured?	1.9±0.6 (1.8-2.1)	2.4±0.8 (2.1-2.8)	<0.05
Know how often blood levels of VAN should be taken once the patient has reached therapeutic range?	2.5±0.9 (2.2-2.9)	2.1±0.6 (2.0-2.3)	0.02
Know the target therapeutic range for VAN?	2.0±0.7 (1.8-2.2)	2.3±0.9 (1.9-2.7)	0.13
Interpret high or low VAN levels to use that information to amend the dose or interval?	2.5±1.1 (2.1-2.9)	2.1±0.8 (1.8-2.3)	0.05
Manage an infusion-related reaction to VAN (red man syndrome)?	3.4±1.3 (2.8-3.9)	3.1±1.0 (2.8-3.4)	0.28

Values are presented as mean ± standard deviation (95% confidence interval). Likert score: 1 = strongly agree, 2 = agree, 3 = not sure, 4 = disagree, 5 = strongly disagree.

VAN, vancomycin.

a) Attendance versus non-attendance, using the unpaired t-test.

**Table 4.** Comparison of self-reported confidence scores between those with and without pocket guidelines (late in training year group only)

Confidence domains regarding vancomycin “do you feel confident to”	JMO without pocket guidelines (n = 17)	JMO with pocket guidelines (n = 58)	P-value^a)^
Treat patients with VAN?	2.9±0.9 (2.4-3.3)	2.1±0.6 (1.9-2.2)	< 0.001
Choose an initial VAN dose?	2.3±0.7 (1.9-2.6)	1.8±0.6 (1.6-2.0)	0.01
Choose a maintenance VAN dose	2.6±0.8 (2.2-2.9)	1.9±0.7 (1.7-2.1)	< 0.001
Know when the first blood level of VAN should be measured?	2.4±0.8 (2.0-2.8)	2.0±0.6 (1.9-2.2)	0.06
Know how often blood levels of VAN should be taken once the patient has reached therapeutic range?	2.6±0.7 (2.1-3.0)	2.2±0.7 (2.0-2.3)	0.03
Know the target therapeutic range for VAN?	2.3±0.8 (2.0-2.7)	2.0±0.8 (1.8-2.2)	0.13
Interpret high or low VAN levels to use that information to amend the dose or interval?	2.7±1.1 (2.1-3.3)	2.1±0.8 (1.8-2.3)	0.01
Manage an infusion-related reaction to VAN (red man syndrome)?	3.0±1.1 (2.7-3.2)	3.8±1.2 (3.2-4.4)	< 0.01

Values are presented as mean ± standard deviation (95% confidence interval). Likert score 1 = strongly agree, 2 = agree, 3 = not sure, 4 = disagree, 5 = strongly disagree.

VAN, vancomycin.

a) Using the unpaired t-test.

**Table 5.** Demographics of respondents (n = 85) who completed the online VAN continuing medical education knowledge assessment

Medical officer characteristic	No. of all respondents (%)
Sex (female)	46 (54.1)
Hospital position	
PGY1	53 (62.4)
PGY2	14(16.5)
PGY3	10(11.8)
Other	8 (9.4)
Hospital	
Flinders Medical Centre	66 (77.6)
Repatriation General Hospital	13(15.3)
Noarlunga Health Service	6(7.1)
How many times prescribed VAN	
≤ 10 times	57 (67.1)
11-20 times	16(18.8)
21-30 times	5(5.9)
> 30 times	5(5.9)
Missing	2 (2.3)
Attended an educational session on vancomycin earlier in the year	45 (52.9)
Possess pocket vancomycin guidelines	58 (68.2)

VAN, vancomycin; PGY, postgraduate year.

**Table 6.** Number and percentage of correct scores attained by respondents (n = 85) completing the online continuing medical education knowledge assessment

Knowledge questions on VAN from clinical vignettes	All	Prescribed VAN ≤10 times (n = 57)	Prescribed VAN >10 times (n = 26)	P-value^a)^	Did not attend prior educational session (n = 40)	Attended prior educational session (n = 45)	P-value^a)^	No pocket guidelines (n = 27)	Possession of pocket guidelines (n = 58)	P-value^a)^
What loading dose would you prescribe?	75 (88.2)	55 (93)	20(77)	0.06	37 (92.5)	38 (84.4)	32	22 (81.5)	53(91.4)	0.28
What subsequent maintenance dose would you prescribe?	77 (89.4)	51 (86)	25 (96)	0.26	38 (95.0)	38 (84.4)	0.16	24 (88.9)	52 (89.7)	1.00
Before which dose should you take the first VAN level?	81 (953)	55 (93)	26(100)	31	39 (97.5)	42 (93.3)	0.62	26 (96.3)	55 (94.8)	1.00
If the patient had a creatinine clearance of 30 mlVmin, before which dose would you take the first VAN level?	77 (89.4)	51 (86)	23 (88)	1	32 (80.0)	42 (93.3)	0.1	21 (77.8)	53 (91.4)	0.10
What is the therapeutic range (intermittent infusion) for vancomycin recommended by the pathology laboratory?	81 (953)	55 (93)	26(100)	31	38 (95.0)	43 (95.6)	1	26 (963)	55 (94.8)	1.00
A patient's initial VAN levels returns as 23.2 mg/L; what dosing regimen will you prescribe?	70 (82.4)	47 (80.0)	23 (88.5)	0.54	36 (90.0)	34 (75.6)	0.1	20 (74.0)	50 (86.2)	0.15
When should the patient's next VAN trough level be checked?	77 (89.4)	53 (89.8)	24 (92.3)	1	37 (92.5)	40 (88.9)	0.72	24 (88.9)	53(91.4)	0.71
How often should a VAN level be measured once the patient is in the target range (provided stable renal function)?	79 (92.9)	54(91.5)	25(96.1)	0.66	38 (95.0)	41 (91.1)	0.68	26 (96.3)	53 (91.4)	0.66
If a patient develops red man syndrome, what changes will you make to the VAN infusion rate?	51 (60)	36 (61.0)	14(53.8)	0.63	25 (62.5)	25 (55.6)	0.66	14(51.9)	36 (62.1)	0.48
How many consecutive VAN levels within the target range will the patient require prior to discharge home for outpatient antimicrobial therapy with VAN?	65 (76.5)	46 (78.0)	18(69.2)	0.42	30 (75.0)	34 (75.6)	1	20 (74.0)	44 (75.9)	1.00

Values are presented as number (%). Likert score: 1 = strongly agree, 2 = agree, 3 = not sure, 4 = disagree, 5 = strongly disagree.

VAN, vancomycin.

a) Using the 2-sided Fisher exact test.

## Supplementary Materials

Supplement 1. Self-confidence survey.

Supplement 2. Vancomycin continuing medical education with knowledge assessment.

Supplement 3. Vancomycin pocket guidelines.

Supplement 4. Data file is available from https://doi.org/10.7910/DVN/GLCXW5.

Supplement 5. Audio recording of the abstract.

## References

[1] Ross S, Maxwell S (2012). Prescribing and the core curriculum for tomorrow’s doctors: BPS curriculum in clinical pharmacology and prescribing for medical students. Br J Clin Pharmacol.

[2] Pillans P (2009). How prepared are medical graduates to begin prescribing?. Intern Med J.

[3] General Medical Council (2014). Be prepared: are new doctors safe to practise? [Internet]. http://www.gmc-uk.org/Be_prepared___are_new_doctors_safe_to_practise_Oct_2014.pdf_58044232.pdf.

[4] Hilmer SN, Seale JP, Le Couteur DG, Crampton R, Liddle C (2009). Do medical courses adequately prepare interns for safe and effective prescribing in New South Wales public hospitals?. Intern Med J.

[5] Avent ML, Vaska VL, Rogers BA, Cheng AC, van Hal SJ, Holmes NE, Howden BP, Paterson DL (2013). Vancomycin therapeutics and monitoring: a contemporary approach. Intern Med J.

[6] Roberts JA, Norris R, Paterson DL, Martin JH (2012). Therapeutic drug monitoring of antimicrobials. Br J Clin Pharmacol.

[7] Rybak MJ, Rotschafer JC, Rodvold KA (2013). Vancomycin: over 50 years later and still a work in progress. Pharmacotherapy.

[8] Confederation of Postgraduate Medical Education Councils (2012). Australian curriculum framework for junior doctors version 3.1 [Internet]. http://curriculum.cpmec.org.au/index.cfm.

[9] Ross S, Loke YK (2009). Do educational interventions improve prescribing by medical students and junior doctors?: a systematic review. Br J Clin Pharmacol.

[10] Davey P, Marwick CA, Scott CL, Charani E, McNeil K, Brown E, Gould IM, Ramsay CR, Michie S (2017). Interventions to improve antibiotic prescribing practices for hospital inpatients. Cochrane Database Syst Rev.

[11] Morrison AP, Melanson SE, Carty MG, Bates DW, Szumita PM, Tanasijevic MJ (2012). What proportion of vancomycin trough levels are drawn too early?: frequency and impact on clinical actions. Am J Clin Pathol.

[12] Alexander C, Millar J, Szmidt N, Hanlon K, Cleland J (2014). Can new doctors be prepared for practice?: a review. Clin Teach.

[13] Tallentire VR, Smith SE, Skinner J, Cameron HS (2012). The preparedness of UK graduates in acute care: a systematic literature review. Postgrad Med J.

[14] Phillips CJ, Wisdom AJ, Eaton VS, Woodman RJ, McKinnon RA (2016). The impact of a pilot continuing professional development module on hospital pharmacists’ preparedness to provide contemporary advice on the clinical use of vancomycin. Springerplus.

[15] Monrouxe LV, Grundy L, Mann M, John Z, Panagoulas E, Bullock A, Mattick K (2017). How prepared are UK medical graduates for practice?: a rapid review of the literature 2009-2014. BMJ Open.

